# Interventions targeting young people not in employment, education or training (NEET) for increased likelihood of return to school or employment—A systematic review

**DOI:** 10.1371/journal.pone.0306285

**Published:** 2024-06-27

**Authors:** Tonje Holte Stea, Thomas Bjerregaard Bertelsen, Liv Fegran, Ellen Sejersted, Annette Løvheim Kleppang, Tonje Fyhn

**Affiliations:** 1 Department of Health and Nursing Sciences, University of Agder, Kristiansand, Norway; 2 Department of Child and Adolescent Mental Health, Sorlandet Hospital, Kristiansand, Norway; 3 The Library, University of Agder, Kristiansand, Norway; 4 NORCE Norwegian Research Centre, Bergen, Norway; Bar-Ilan University, ISRAEL

## Abstract

The present systematic review aims to identify, synthesize, and evaluate evidence of effects from interventions targeting youth not in education, employment, or training (NEET). We searched relevant multidisciplinary databases to identify randomized controlled trials (RCTs) and quasi-randomized re-engagement trials. Primary outcomes were participation in education and employment, and training status. Secondary outcomes included changes in financial status, quality of life and well-being, social functioning, and physical, psychological, and behavioral outcomes. The Joanna Briggs Institute methodology and PRISMA guidelines were applied. Eligible studies were screened, included, and extracted for data. Nine trials were included (eight RCTs and one quasi-experimental study), in which samples ranged from 96 to 7346 participants. Results on primary outcomes showed that five studies found an effect of interventions on employment outcomes, while three studies indicated an effect on education or training. Results on secondary outcomes included effects on mental health, subjective health complaints, drug use, self-esteem, and self-efficacy. Studies with other main outcomes than re-engagement showed an effect of interventions on pass rates for the driving test, independent housing, and increased job-seeking activities. Limitations and future directions are discussed, including the lack of rigorous studies, theoretical underpinnings, and standardized measures for re-engagement outcomes.

**Systematic review registration:** registered in PROSPERO, CRD42023463837.

## Introduction

Young people who are Not in Education, Employment, or Training (NEET) represent a societal concern worldwide [1]. The term NEET is used as a broad category covering a heterogeneous population of young people aged between 15 and 29 and is associated with a range of life difficulties [2]. Being identified as NEET is associated with poorer health and well-being in the short- and long term and also has a negative impact on the economic growth and welfare of countries [3–6]. Previously, studies have reported that NEET status is associated with increased risk behaviors [7, 8], poorer self-perceived health and poorer mental health, and increased risk of injury due to self-directed violence and violence from others [3, 9–11] compared to their non-NEET peers. A systematic review and meta-analysis confirmed that being in the NEET category was associated with substance use and severe mental health problems and that mental health problems in early youth predicted later status as NEET, while evidence for the inverse relationship was equivocal and sparse [12]. Moreover, results from primary care services have shown that young NEET adults seeking psychological treatment for common mental disorders had worse treatment outcomes than young adults who were not NEET [13]. As expected, studies have documented lower socioeconomic status among those in the NEET population compared to their peers in school, which may partly explain differences in health status [10, 14].

Recently, there has been a major concern about how young people will be affected by the economic consequences of COVID-19 [15–17]. Globally, the COVID-19 pandemic had particularly severe and wide-ranging impacts on young people, which is reflected by NEET rates of 24.9% in 2020 and 23.5% in 2022, well above their pre-pandemic levels [18]. EU countries have also reported an increase in NEET rates during the pandemic, but the long-term trend is a significant decrease during the last decade, falling from 16.0% in 2012 to 11.7% in 2022 [19]. Nevertheless, reducing the NEET rate to 9% by 2030 is one of the targets of the European Pillar of Social Rights [20]. According to the Sustainable Development Goals (SDGs) 8.6, the proportion of youth (aged 15–24 years) not in employment, education, or training should have been substantially reduced worldwide by 2020 [21].

Although NEET rates have been reduced in EU countries, it is still considered a significant challenge because of the risk of health deterioration posed by this state [4], its long-term "scarring” effect [22], as well as the labor market’s unmet supply of workers due to an ageing working population [23].

To ensure effective outreach to young people in the NEET category, it is highly important to use measures which are designed and tailored to their background and needs. Worldwide, NEET rates vary according to regional affiliation and individual background characteristics. Two thirds of NEETs are women; the NEET rates and gender gap is higher in rural compared to urban areas; higher among certain ethnic minorities; and NEET rates are inversely related to country income and individual educational attainment [18, 24–27]. Vulnerable NEETs with serious mental health conditions who are disengaged and not actively looking for work and/or training opportunities, may also experience other challenges, which often require special outreach activities for psychiatric rehabilitation [28]. Furthermore, lessons from the COVID-19 pandemic have shown that governments must invest in social safety net programs that focus on supporting those most at-risk [29]. The International Labour Organization identified a total of 73 countries as having widespread social partner engagement in their national youth strategy across several stages of youth employment policymaking [30]. Longitudinal analyses based on data from Eurostat and World Bank have also demonstrated that the measures targeting NEETs in order to facilitate access to education, the labor market, and social inclusion, must be coordinated with measures alleviating poverty and any type of exclusion, the support given to employers (i.e., subsidizing jobs), the family and the local community [31].

According to the literature, a range of different approaches have been used to increase the likelihood of youth to return to education, employment or training, including financial support, intensive support from trained advisors, and tailored education, employment and training solutions [32]. A systematic review and meta-analysis of re-engagement interventions for the NEET population concluded in 2017 that there was a need for research to adopt high-quality evidence methodologies to determine what works best for this population [33]. A recently published evidence and gap map based on systematic reviews and impact evaluations concluded that most youth employment interventions originate from high-income countries, have an experimental study design, and provide low quality evidence [34]. Thus, due to the limited knowledge base for developing recommendations for policy and practice, it is crucial to continually identify programs using methods and strategies that effectively contribute to re-activate NEETs into employment, education and/or training. The present systematic review seeks to address these gaps by identifying, synthesizing, and evaluating evidence of effects from randomized and quasi-randomized re-engagement interventions targeting NEETs.

## Methods

To ensure a rigorous review process, we followed the guidelines from the Joanna Briggs Institute [35]. The protocol for the review was registered in the PROSPERO database 25. September 2023 (record ID 463837). The principles underlying the Preferred Reporting Items for Systematic Reviews and Meta-Analyses (PRISMA 2020) framework for data retrieval, were adopted in terms of identification, screening, eligibility, and inclusion [36]. The PRISMA 2020 checklist is available as S1 File.

### Eligibility criteria

Eligibility criteria were constructed around population, intervention, comparison, and outcomes (PICO) [35].

**P_**The population of interest was young people aged between 15 and 29 years who were not in employment, education, or training (NEET) at the time of recruitment. We also included studies where the sample age range exceeded that stated above, if the study reported separate results for the age group 15–29 years. No restrictions according to regional affiliation were applied. Studies including both NEET and non-NEET groups were excluded if intervention effects were not reported separately for the NEET group.

**I_** All interventions targeting the NEET population, such as group-based or individual counselling, educational and training programs, financial and career development programs, structured social interventions, and multi-component interventions were included.

**C**_Only randomized and quasi-randomized trials with a concurrent or counterfactual control group and baseline equivalence were included. For quasi-randomized studies, baseline equivalence or use of a valid matching procedure was ensured. Studies were also included if comparison groups received treatment as usual or were assigned as waiting-listed controls. Studies with the following design were not included: cross-sectional studies, pre-post studies, and studies with non-comparison groups.

**O_** Primary outcomes for the review were school attendance and employment, i.e., participants are classified as engaged (in education, employment, or training) or not engaged (not in employment, education, or training). Thus, the review included studies reporting data from a valid assessment of education and employment and training status at baseline and after the intervention period. As secondary outcomes in the review we included studies that report changes in e.g., financial status, quality of life and well-being, social functioning, as well as physical, psychological, and behavioral outcomes.

### Information sources and search strategy

The search strategy was designed to find primary studies published in journals on the effects of any type of intervention, including control groups, and outcomes for the NEET population. Studies from grey literature were not included. The following search elements were used: words for the NEET population and intervention. Text-word and index-word, if applicable, were applied. From the preliminary test searches in several reference databases (the core collection of Web of Science, Scopus, MEDLINE and EMBASE, together with Google and Google Scholar) and by reading the literature, many different phrases were used for people with NEET status and consequently used in the search strategy. Other phrases that we were not aware of were covered by searching words nearby each other by using proximity operators, like ADJn used in Ovid and NEAR/n in WoS (Web of Science). It is possible that some studies would not use words for NEET status in the title or abstract but still include people with NEET status; thus, by searching the title for words for youth and employment, unemployment, or similar words, more relevant studies could be retrieved. We searched the following databases, on the 25^th^ and 28^th^ of August 2023: MEDLINE, EMBASE, APA PsycInfo and Cochrane Central Register of Controlled Trials simultaneously through Ovid, WoS (from the Core Collection, the following databases: Science Citation Index Expanded, Sciences Citation Index, Emerging Sources Citations Index), Scopus, SocINDEX (EBSCO*host*), ERIC (EBSCO*host*) and CINAHL (EBSCO*host*). The searches were updated on November 28, 2023, and executed the same way as the original, with an additional term for the different spelling of labour (labor). Full search strategies from all databases from the updated searches are attached as S2 File.

The reference lists of the included studies were manually screened during full-text reading. Furthermore, the review conducted by Mawn et al. [33], which had similar inclusion and exclusion criteria as our study, were screened for additional studies.

### Study selection

Searches in databases via Ovid were conducted simultaneously, and duplicates were automatically removed through the search. Subsequently, all hits from the searches were exported to EndNote 21 to eliminate the remaining duplicates before screening (ES) by comparing different field values in a stepwise process inspired by Bramer et al. [37]. Finally, some duplicates were identified after importing the records to Rayyan, a web tool designed to help researchers expedite the initial blinded screening of abstracts and titles [38]. Titles and abstracts were screened independently in Rayyan by two teams of two reviewers (THS/TF/LF and LF/ALK) against the inclusion criteria. To ensure quality in the screening process of titles and abstracts, both teams of reviewers screened 10% randomly selected, identical reports based on the predefined inclusion and exclusion criteria. During the study selection process, discrepancies, and disagreements within a pair of reviewers were resolved through discussion or, when necessary, by involving the other team of reviewers. When the inclusion or exclusion of a report was not possible to determine based on the information in the title and abstract, a full-text screening of the report was conducted. In cases where full-text screening did not provide sufficient information on the inclusion criteria, authors were contacted directly by e-mail.

### Quality assessment

Two teams of two reviewers (ALK/TBB, THS/TBB) independently assessed the methodological quality of each report, including adequacy of randomization, allocation concealment, blinding, and completeness of follow-up. They worked independently and used the risk of bias assessment tool published by JBI, Critical appraisal checklist for randomized controlled trials and quasi-experimental studies [35], to assess each domain as adequate, unclear, or inadequate. Disagreements were resolved by discussion or by the project leader. Assessment of study quality and data extraction information are reported in a tabular format.

### Data extraction

Two teams of two independent reviewers (TF/ALK, THS/TB) conducted data extraction from the selected studies using a structured template developed by the authors, which was based on the quality assessment tools. The template included the following data:

1. Background information: publication year, authors, title, country, aim of the study, study funding sources.
2. Methods: study design, recruitment and selection of the participants, theoretical basis, level, intervention components of the intervention and control programs, duration of follow-up.
3. Sample characteristics: n, sex, age, socioeconomic status, type of youth population, length of NEET status pre-intervention, behavior- and health characteristics pre-intervention.
4. Comparators: time between pre-treatment and assessment.
5. Outcomes: Data extraction of outcomes will follow the procedures outlined by JBI. Primary outcomes for the review are school attendance and employment, i.e., assessments of status will include those in which individuals are classified as engaged (if they are in education, employment, or training) or not engaged. Other outcomes may include health-related behaviors, antisocial behaviors, physical and mental health, quality of life, well-being, earnings, welfare receipt, which are described as secondary outcomes in the review.
6. Process: response rate, study completion rates, costs.

### Data synthesis

Findings from the included studies were synthesized based on results from the critical appraisal process and the results from the data extraction. According to the protocol, we intended to conduct a systematic review and if possible, a meta-analysis, of interventions targeting NEETs. However, the data extraction process revealed that there was not sufficient homogeneity in the types of intervention and/or study designs included to conduct a meta-analysis.

**Fig 1:**
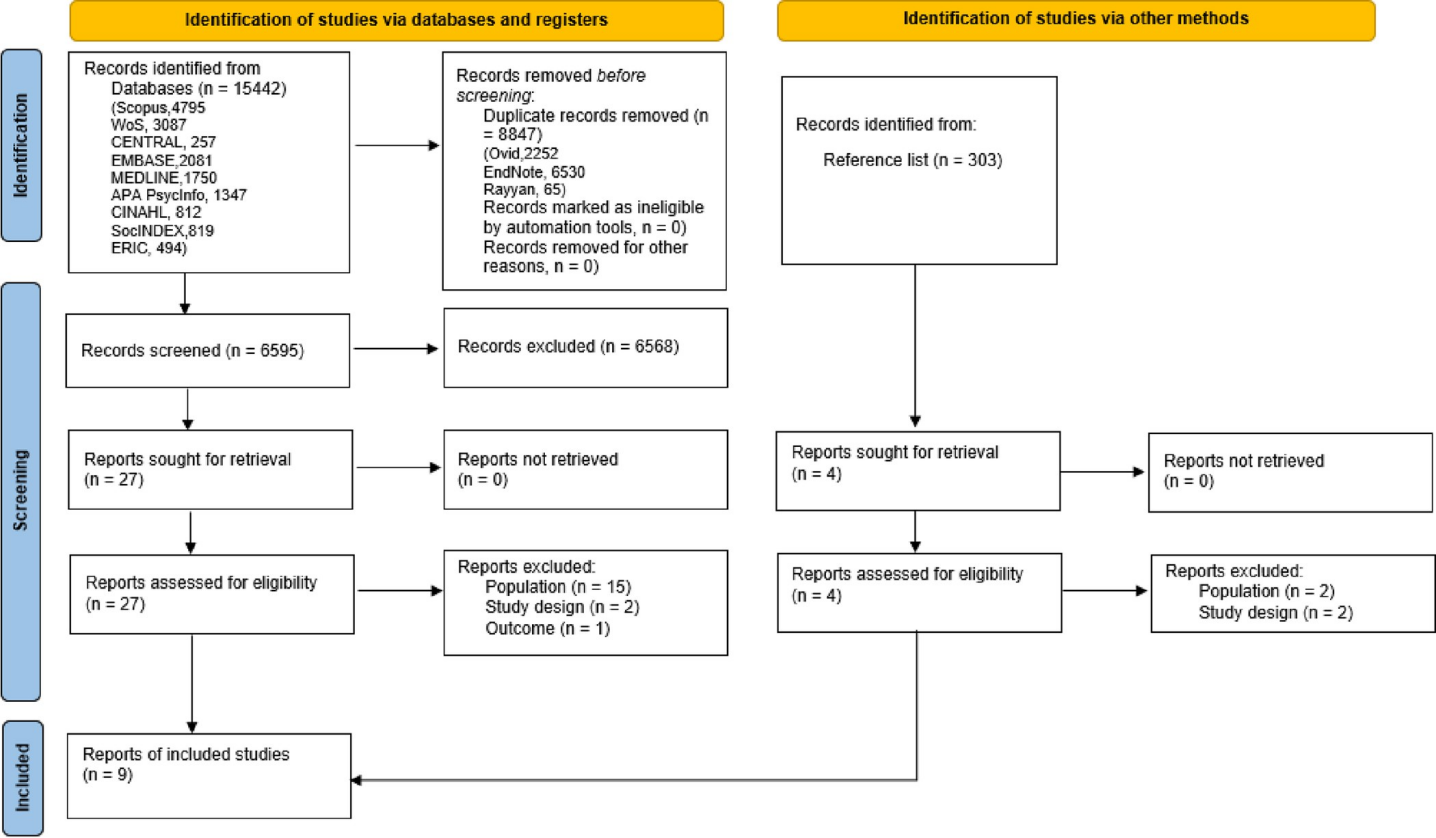
PRISMA 2020 flow diagram.

Search results, screening outcomes, and selection decisions have been documented and presented in a PRISMA 2020 flow diagram format for systematic reviews (Fig 1).

## Results

Database searches identified 15442 records. Some duplicates (n = 2252) were removed automatically through Ovid; the rest of the duplicates (n = 6530) were removed through EndNote 21. Additional duplicates were found through the screening process in Rayyan (n = 65). In total, 6595 records were screened by title and abstract. A total of 6567 records were excluded during the screening of titles and abstracts. Full-text versions of the remaining 27 papers were obtained and assessed against the inclusion criteria. Of these, 18 papers did not meet the inclusion criteria and were excluded. In addition, full-text versions of four papers after manually screening of the review by Mawn et al. [33] were obtained and assessed against the inclusion criteria. All these papers were excluded.

Reasons for exclusion is presented in S3 File. A total of 9 articles were included after thorough evaluation of methodological quality (Tables 1 & 2).

**Table 1:** Critical appraisal of randomized controlled trials.

Study	Q1	Q2	Q3	Q4	Q5	Q6	Q7	Q8	Q9	Q10	Q11	Q12	Q13	Total yes
Bond et al., 2016 [39]	Y	N	Y	N	N	N	Y	Y	Y	Y	Y	Y	Y	9/13
Card et al., 2011 [40]	Y	N	U	N	N	N	U	Y	U	Y	Y	Y	Y	6/13
Eppel & Mahringer, 2023 [41]	N	N	N	N	N	N	Y	Y	Y	Y	Y	Y	Y	7/13
Le Gallo et al., 2016 [42]	Y	N	Y	N	N	Y	Y	Y	U	Y	Y	Y	Y	9/13
Jensen et al., 2003 [43]	N	N	U	N	N	Y	Y	Y	Y	Y	Y	Y	Y	8/13
Patel et al., 2020 [44]	N	N	N	N	N	Y	N	Y	U	N	Y	N	N	3/13
Robert et al., 2019 [45]	Y	N	Y	N	N	Y	U	Y	U	Y	Y	N	N	6/13
Sveinsdottir et al., 2019 [46]	Y	Y	Y	N	N	N	Y	Y	Y	U	Y	Y	Y	9/13

Y, yes; N, no; U, unclear; N/A, not applicable. JBI critical appraisal checklist for randomized controlled trials.

Q1 Was true randomization used for assignment of participants to treatment groups?

Q2 Was allocation to treatment groups concealed?

Q3 Were treatment groups similar at baseline?

Q4 Were participants blind to treatment assignment?

Q5 Were those delivering treatment blind to treatment assignment?

Q6 Were outcome assessors blind to treatment assignment?

Q7 Were treatment groups treated identically other than the intervention of interest?

Q8 Were outcomes measured in the same way for treatment groups?

Q9 Were outcomes measured in a reliable way?

Q10 Was follow-up complete and if not, were differences between groups in terms of their follow up adequately described and analyzed?

Q11 Were participants analyzed in the groups to which they were randomized?

Q12 Was appropriate statistical analysis used?

Q13 Was the trial design appropriate, and any deviations from the standard RCT design (individual randomization, parallel groups) accounted for in the conduct and

analysis of the trial?

**Table 2:** Critical appraisal of quasi-experimental studies.

Study	Q1	Q2	Q3	Q4	Q5	Q6	Q7	Q8	Q9	Total yes
Ehlert et al., 2012 [47]	Y	N	Y	Y	Y	U	Y	Y	Y	7/9

Y, yes; N, no; U, unclear; N/A, not applicable.

JBI critical appraisal checklist for quasi-experimental studies

Q1 Is it clear in the study what is the ‘cause’ and what is the ‘effect’ (i.e., there is no confusion about which variable comes first)?

Q2 Were the participants included in any comparisons similar?

Q3 Were the participants included in any comparisons receiving similar treatment/care, other than the exposure or intervention of interest?

Q4 Was there a control group?

Q5 Were there multiple measurements of the outcome both pre and post the intervention/exposure?

Q6 Was follow-up complete and if not, were differences between groups in terms of their follow-up adequately described and analyzed?

Q7 Were the outcomes of participants included in any comparisons measured in the same way?

Q8 Were outcomes measured in a reliable way?

Q9 Was appropriate statistical analysis used?

### Methodological quality

Eight of the included studies were RCTs and were critically appraised using the JBI tool for RCTs. The study by Ehlert and co-workers [47] had a quasi-experimental design and was critically appraised using the JBI tool specifically developed to evaluate studies with this design [35]. The methodological quality of the RCT studies ranged from three points [44] to nine points [39, 42, 46]. A few patterns in the critical appraisal table are worth noting. Half of the RCT studies did not report conducting true randomization to assign participants to treatment groups (Q1), or did not report adequately on this criterion, which is likely to have resulted in unsimilar treatment groups at baseline in these same studies (Q3). The lack of blinding (Q4, Q5) in randomization and treatment delivery is not surprising, given the psychosocial nature of the interventions. All studies measured outcomes in the same way across treatment groups (Q8), however, only four studies used reliable outcome measures (Q9). Eppel and Mahringer [41] and Jensen et al. [43] used register data to measure outcomes, while the IPS studies conducted by Bond et al. [39] and Sveinsdottir et al. [46] used the same standardized self-report measure of employment. Nearly all RCTs adequately reported to use appropriate procedures for statistical analyses (Q11, Q12), and trial design (Q13). Overall, most of the included RCTs were of adequate or good quality. This also applies for the quasi-experimental study by Ehlert et al., [47] which scored 7/9 on the criteria for this type of study.

### Background information

Only information related to background, methods, sample characteristics comparators and process described in the template for data extraction, which was provided by all included studies, is reported in Table 3.

**Table 3:** Background information about study sample, study design, program components and outcome measures.

Author, Year, Country	Study sample	Study design	Program	Outcomes
**Bond** et. al., 2016 [39] USA	I-group: n = 49, 67% M C-group: n = 60, 70% M Age: 20–29 years	18 months RCT	The Individual Placement and Support (IPS) model for individuals with severe mental illness across four US locations compared to Treatment As Usual (TAU)	**Primary outcomes:** Competitive employment outcomes
**Card** et al., 2011 [40] Dominican Republic	I-group: n = 5723, 55% F C-group: n = 1623, 58% F Age: 16–29 years	1–18 months (median 13 months) RCT	Basic skills training to strengthen self-esteem and work habits, and technical/vocational training at local employers, customized to employer needs. Treatment compared to waiting list controls	**Primary outcomes:** Employment outcomes and labor market earnings
**Eppel & Mahringer**, 2023 [41] Austria	I-group: n = 977, 39% F C-group: n = 834, 30% F Age: 15–21 years	17 months RCT (cluster-randomized)	Intensified support by case managers with extra coaching of individuals compared to TAU	**Primary outcomes:** Counseling and placement process outcomes and integration into training, education, and employment
**Le Gallo** et al., 2016 [42] France	I-group, n = 1000, 56% F C-group, n = 1000, 57% F Age: 16–25 years	1–2 years RCT	Financial and non-monetary aid providing vouchers for driver’s licenses to individuals compared to TAU	**Primary outcomes:** Mobility indicators including driving, housing, and employment status
**Jensen** et. al., 2003 [43] Denmark	I-group, n = 1166, 49% F C-group, n = 1808, 51% F Age: 16–24 years	18 months RCT	’Youth Unemployment Program’ (YUP) providing added value due to a political reform focusing on benefits, incentives and sanctions, compared to TAU	**Primary outcomes:** Transition from unemployment to schooling and employment
**Patel** et. al., 2020 [44] South Africa	I-group, n = 1003, 39% M C-group, n = 889, 38% M Age: 18–25 years	12 months RCT (cluster-randomized)	Youth Employability Programs (YEPs) plus money management focusing on employability training and coaching, bolstered by a financial capability curriculum compared to traditional YEP (TAU)	**Primary outcomes:** Self-esteem, self-efficacy and future orientation and job-search resilience
**Robert** et. al., 2019 [45] France	I-group, n = 504, 50% F C-group, n = 472, 54% F Age: 18–25 years	12 months RCT	Social and preventive medicine consultation with a social worker and MD to increase access to training compared to TAU provided by social services	**Primary outcomes:** Participation in training sessions. **Secondary outcomes:** Access to employment and health status
**Sveinsdottir** et. al., 2019 [46] Norway	I-group, n = 50, 64% F C-group: n = 46, 72% F Age: 18–29 years	12 months RCT	Comparing the IPS model for individuals on long-term health benefits to TAU providing traditional vocational rehabilitation	**Primary outcome:** Paid employment. **Secondary outcomes:** Physical and mental health, well-being, coping, alcohol consumption and drug use
**Ehlert** et al., 2012 [47] Germany	I-group: n = 211, 16% F C-group: n = 103, 27% F Average age: 23 years	12 months Quasi-experimental study	Individual coaching, classroom training and temporary work compared to waiting list controls	**Primary outcomes:** Employment probability (employment and unemployment history, earnings, occupation, some firm information, educational attainment, active labor market program participation)

### Synthesis of study results

#### Employment

In the identified studies, various outcomes related to employment were assessed, with a notable emphasis on the effectiveness of different interventions. Bond et al. [39], reported that the IPS model significantly improved self-reported employment outcomes with effect sizes ranging from medium (0.48) to large (0.86) (*p*<0.001), where 82% of participants in the intervention group acquired a job compared to 42% in the control group. Similar positive outcomes were observed for ‘ever working >20 hours’, ‘total weeks worked’, ‘job tenure weeks’, ‘total hours worked’, ‘hours per week’, ‘total wage earned’, ‘number of jobs held’, and ‘days to first job’, all showing substantial effect sizes and statistical significance (*p*<0.001). The IPS study by Sveinsdottir et al. [46], also found a stark contrast between groups on the self-reported outcome ’Competitively employed’ (Cohen’s *d* = 0.96, *p* = 0.001), with 48% being employed in the intervention group (IPS) versus 8% in the control group (conventional work program), as measured by register data. Similar significant outcomes were observed for the self-reported outcomes ’Ever worked > 20h’ and ’Hours worked’, with substantial effect sizes and statistical significance, favouring the intervention group. In contrast, the youth training program evaluated by Card et al. [40] showed no significant effect on self-reported employment, and only a modest (10%) impact on earnings per month (conditional on employment), although the estimated effect was only marginally significant (*t* = 1.5). Similarly, Eppel and Mahringer [41] reported that intensified support for NEETs did not have a significant impact on the overall extent of integration into employment as measured by register data, after the three-year follow-up period. The study by Le Gallo et al. [42] showed no significant differences in self-reported employment status at 12 and 24 months after focusing on improving participants’ mobility. Effects from the Youth Unemployment Program combining benefits, incentives and sanctions to reduce unemployment duration and increase transition rates from unemployment to schooling and employment (register data), reported by Jensen et al. [43], indicated a better hazard rate at 9 months, although the results were somewhat unclear given the relatively low sample size. In the social and preventive medicine consultation intervention study by Robert et al. [45], no significant difference in working status was observed between participants in the intervention group (78% worked) compared to the control group (76% worked) after the intervention period. In the study by Ehlert et al. [47], no significant effect on employment outcomes were found for either short (6 months) nor medium (12 months) durations. Although subgroup analyses indicated that participants who stayed in the program for the longest duration (6–12 months) had higher chance of being employed after 6 and 18 months, respectively, these results do not take into account the randomized design and therefore do not provide conclusive evidence.

#### Education and training

Jensen et al. [43] and Robert et al. [45] assessed the effectiveness of specific interventions on outcomes related to education and training, in addition to employment outcomes. The study by Jensen and co-workers [43] focused on the effect of the Youth Unemployment Program (YUP) on youth’s transition from unemployment to school or training. Findings show increased hazard rates for this transition in the YUP group at 6 and 9 months, but the benefits were not observed beyond these time points. On the other hand, the study by Robert and co-workers [45] examined participation in training programs as the primary outcome, reporting increased participation rate for the intervention group compared to the control group (63% vs. 56%, p = 0.039). The positive effect was only significant for specific sub-groups, including females (p = 0.003), those aged ≤21 years (p = 0.041), those with unstable housing (p = 0.013) and low and medium low educational level (p = 0.03 and p = 0.045, respectively). Likewise, Eppel and Mahringer [41] reported an increase in having received basic training (p<0.01) when comparing active case management (46%) with no case management (35%), but no improvement on further training or education was observed.

#### Outcomes that may increase probability of employment

Le Gallo et al. [42] and Patel et al. [44] assessed the effectiveness of various interventions which did not directly measure the impact on employment or enrolment status, but rather on indicators which may increase probability of employment. The study by Le Gallo and co-workers [42] revealed that 53% of intervention participants passed the driving theory test at 12 months, compared to 34% in the control group (*p*<0.001). For the practical test at the same interval, 48% passed versus 40%, with a smaller but still significant difference (*p* = 0.039). However, the total number of driving hours at 12 months showed no significant difference. At 24 months, the pass rates increased significantly for the theoretical test (I:66% C:47%, p = <0.001) and the practical test (I:68% C:63%, *p* = 0.157). The total driving hours at 24 months increased during this period (24.63 vs 32.21, *p<*0.001). Independent housing (not living with parents) increased among the intervention group compared to the control group at 12 months *(p =* 0.01), and at 24 months (*p =* 0.05). The intervention studied by Patel et al. [44] resulted in increased job-seeking activities, such as more individuals searching for job ads (57% vs. 68%), visiting employment agencies (20% vs. 35%), enquiring with employers (19% vs. 30%), placing ads (16% vs. 32%), searching assistance from friends and family (15% vs. 30%), searching financial assistance (77% vs. 10%), trying to start a business (3% vs. 9%), and waiting where casual workers are hired (2% vs. 6%).

### Secondary outcomes

In addition to primary employment and educations metrics, the reviewed studies also explored a variety of secondary outcomes and conducted subgroup analyses. Of notice, Bond et al. [39] found IPS to be effective for both younger (under 25) and older adults (25–29) in terms of employment-related outcomes. In terms of secondary outcomes Sveinsdottir et al. [46] found that the IPS group improved significantly more on measures of mental health, subjective health complaints and drug use. Patel et al. [44] found that the intervention group (additional financial management) maintained their future outlook based on measures of self-esteem and increased self-rated self-efficacy compared to a possibly more detrimental trend observed in the control group during the intervention period. Robert et al. [45] did not find any improvement in terms of perceived overall health in the intervention group compared to the control group.

## Discussion

The present systematic review provides information about the effectiveness of interventions with a randomized controlled design targeting young adults not in education, employment, or training (NEET). The papers included did not provide sufficient data to conduct a meta-analysis. Studies included in the present systematic review reported outcomes of employment, education, or training, as well as factors that may facilitate transition to these states. Findings in the included studies reported various types of interventions, characteristics of target groups, regional affiliation, and degrees of effectiveness on outcomes.

The Individual Placement and Support (IPS) model demonstrated notable effectiveness in enhancing job acquisition and stability for individuals with severe mental illness [39] and those on long-term health benefits [46]. This consistency in effectiveness, including improved mental health and decreased health complaints and drug use, was observed across various age groups. While IPS aids young adults with serious mental illness in securing stable employment, its generalizability beyond patient populations needs further exploration [48].

Contrasting with IPS, other interventions presented varied outcomes. For instance, the approach by Robert et al. [45] focusing on social and preventive medicine consultations, showed moderate improvements in work status and training program participation, particularly benefiting specific subgroups such as younger adults and those with lower educational levels. Similarly, the study by Ehlert and co-workers [47], indicated that an innovative combination of individual coaching, classroom training and temporary work program was successful in increasing employment rates after 6 and 18 months, but only for participants who stayed on during the complete program. In line with these results presented in the current review, Mawn and coworkers [33] have previously emphasized the importance of program adherence, using a sufficient ‘intervention dose’ and concluded that multi-component interventions seemed to increase employment rates more effectively than single-component interventions.

In contrast, the youth training program by Card et al. [40] focusing on basic skills and technical/vocational training among NEETs in the Dominican Republic showed no significant effect on employment, and only a very modest impact on earnings per month. No sub-group differences in estimated impact on employment were observed according to gender, age education or location, but for those with higher education and living in urban areas, the program seemed to have a positive effect on earnings. Although it has been reported that training impacts in Latin America are on average more positive than those implemented in European countries and the US [49], Card and co-workers conclude that similar programs can most likely not be successfully implemented in Latin American and Caribbean countries due to the many barriers and problems related to financial and operational constraints [40].

Similarly, Eppel and Mahringer [41] reported that intensified support for NEETs did not have a significant impact on the overall extent of integration into employment after the three-year follow-up period. However, their primary objective was to encourage NEETs to pursue education and training to improve their long-term labor market opportunities instead of quickly placing them in random unskilled jobs. Thus, an increase in basic training was observed during the intervention period, but no improvement in further training or education.

The study by Le Gallo et al. [42] focused on improving the degree of mobility among NEETs to avoid social exclusion and improve access to employment, and results indicated only a weak positive effect on access to temporary jobs but not permanent jobs. The high cost and low impact of the program implies that focus on increasing mobility as an approach to increase re-engagement should be re-evaluated or be part of multi-component, cost-effective intervention studies.

Results from the Youth Employability Program (YEP) reported by Patel et al. [44] showed increased job-seeking activities that may be explained by increased job-search resilience, self-esteem, self-efficacy and future orientation, after receiving employability training and coaching with additional focus on increasing financial capability, compared to participation in traditional YEP programs. Based on their findings, Patel and co-workers suggested that future studies should focus on adaptive programming and management [44]. This suggestion is in line with a previous study of re-engagement initiatives for NEETs which emphasized the need of holistic and flexible services to help NEETs gain confidence and competence [50].

Finally, the Youth Unemployment Program (YUP) by Jensen et al. [43], which focused on the use of benefits, incentives and sanctions to decrease youth unemployment, showed only weak short-term effects. Active labour market policies are found in almost all countries worldwide, but varies in amplitude, design and implementation [51]. Results presented by Jensen and co-workers which indicated limited effectiveness in facilitating the transition from unemployment to employment, emphasize the need for further investigation into long-term effects and more adaptive labor market policies as proposed by the European Commission. [52].

The interventions described in this review span simple transactions such as offering vouchers for the driving test [42], changes in active labor market policies [43], intensifying existing counselling practices [41, 44, 45, 47], increasing training [40, 44, 47], and structured, manual-based methodologies requiring cross-sectoral collaboration (IPS) [39, 46]. The two latter studies show the most promising results on re-engagement, but studies are few. Their findings are, however, in line with meta-analyses and reviews of IPS for both adults and young persons with severe mental illness, showing a notable difference between IPS and TAU [48, 53].

The included studies also exemplify the span in approaches within the work rehabilitation field. Re-engagement approaches can broadly be categorized into train-place approaches or place-train approaches [54]. Train-place approaches emphasize stepwise approaches to employment, using unpaid work practice, apprenticeship, or sheltered training to increase participants’ employability. Place-train approaches, on the other hand, emphasize real-world training by pursuing competitive employment based on participant’s goals, while ensuring continual support also after employment has been obtained (ibid). These approaches are present in the included studies in the current review, with the train-place approach exemplified by Card et al. [40] and Eppel and Mahringer [41], while the place-train approach is exemplified in the IPS studies by Bond et al. [39] and Sveinsdottir et al. [46]. Eppel and Mahringer [41], criticize the place-train (or work-first) approach, due to concerns that quickly obtaining employment may channel young people into unsustainable employment, and that this may be avoided by providing training first, in the form of subsidized apprenticeships or qualification measures prior to the job search. In the study by Sveinsdottir et al. [46], on the other hand, sheltered work practice has a much lower success rate than IPS, which is attributed to the lock-in effect of sheltered programs and apprenticeships. Another critique towards using sheltered work practice and apprenticeships is that this benefits the employer, but it does not commit them to engage the young people after the training period is over. This is exemplified in the study by Card et al. [40], where field teams anticipated that nearly all participants were let go from the workplace after the subsidized training period ended.

The widely different approaches to re-engaging NEETs demonstrated in the current review points to a fundamental weakness in the field that needs to be addressed, namely the lack of a guiding theoretical framework for the specific interventions. The absence of theory-driven approaches to increase re-engagement may have limited the methods and strategies used and consequently, possibly limiting intervention effects. This is crucial in the development, implementation, and evaluation of intervention studies, as it provides transparency into intervention components and the linkage between them, as well as enabling learning across contexts.

Another notable weakness across most studies included in this review is the lack of agreement on how to measure reengagement in work or education, and what outcomes are relevant pre-employment measures. Most studies use self-report, and it is unclear how participants were queried re-engagement. This is a threat to both the internal and external validity of the outcome measures.

It is important to note the substantial variation in the methodological quality of the studies reviewed. While some well-documented and robust RCTs were found, it is important to underscore that the majority of the included articles show methodological issues that compromise the validity and generalizability of their findings. Several studies did not conduct true randomization, and instead resorted to related techniques such as propensity score matching. Furthermore, the lack of blinding among participants, practitioners, and researchers to treatment assignment also diminishes the reliability of findings from studies. Compounding these issues, the reliability of assessments was often difficult to evaluate due to the sparse information provided. The findings from this systematic review highlight the critical need for improved methodological rigor. Although implementing robust designs may present ethical and practical challenges, the field would benefit significantly from fewer studies but higher-quality RCTs that incorporate treatment blinding, to the extent possible, and utilize well-documented outcome measures. Indeed, prioritizing the quality over the quantity of studies could transform research outcomes, providing more definitive and generalizable results.

The program and methodological challenges addressed in this review demonstrate the need for different approaches to designing and testing interventions for the NEET group. Innovative approaches such as Sequential Multiple Assignment Randomized Trials (SMARTs)[55], multiple baseline designs [56], and Bayesian sequential testing [57] offer promising avenues for achieving more rigorous RCT designs without imposing excessive burdens on participants or practitioners. SMARTs have been suggested to represent an adaptive intervention approach to design high-quality data after adjusting the type, dosage or delivery mode of an intervention based on participant characteristics and background and make further adjustments according to their ongoing performance, intervention adherence and changing needs [58]. Thus, the SMART involves multiple stages of randomizations and is a design that experimentally assesses the efficacy of the decisions, components, and sequence of an adaptive intervention [59].

### Limitations

In the present study, we only reviewed high-quality evidence based on published studies with a randomized controlled design or a quasi-randomized design with matching control groups. Also, since not sought for grey literature, it is also possible that we may have missed good quality randomized controlled studies not reported in journals. Thus, we may have omitted other studies that could have provided useful and important insight. Qualitative trials, case studies, service evaluations and results from studies that use other designs may contribute to a broader understanding of why different intervention studies fail or succeed in supporting and re-engaging NEETs. Moreover, some of the included studies have acknowledged certain limitations in both the design and conduction of the interventions, including limited sample sizes, somewhat unclear randomization procedures, treatment in control groups, and handling missing data. In view of the heterogeneity among NEETs, studies of interventions tailored to different contexts and targeting sub-groups with different social and educational profiles would have been a strength.

## Conclusion

The studies included in this review show a variety in intervention designs, study designs and outcome measures in studies of interventions targeting young people in the NEET category. The review shows some characteristics of the research and practice field that future intervention developers should make note of. Firstly, there are not many studies employing a rigorous design to test the effect of interventions for this target group, despite the attention that this group receives at the political level. It is worrying that efforts directed towards this group are not tested for effect, given the potential long-term detrimental outcomes of being in this situation. Secondly, the intervention studies included in this review do not seem to be guided by theoretical frameworks. Theoretically underpinned interventions would enable efforts in different contexts to learn from each other, by investigating and discussing how intervention components are linked to each other and to the selected outcomes. Thirdly, there are no standardized outcome measures for re-engagement for the NEET group, which should be the primary target of efforts directed at this group. Future studies should aim to develop interventions and measurements that generalize learning across studies and across contexts.

## Supporting information

S1 File (PDF)

S2 File (PDF)

S3 File (PDF)
