# Supplementary material for: Interventions targeting young people not in employment, education or training (NEET) for increased likelihood of return to school or employment—A systematic review
Source: PLoS One. 2024 Jun 27;19(6):e0306285. doi: 10.1371/journal.pone.0306285 (PMC11210863; doi:10.1371/journal.pone.0306285)
Supplement: S2 File — (PDF) [file pone.0306285.s002.pdf]

WoS, Search date: 28 Nov. 2023

- Science Citation Index Expanded (SCI-EXPANDED, 1945-present)
- Social Sciences Citation Index (SSCI, 1956-present)
- Emerging Sources Citations index (ESCI) 2018-present

Search strategy, will be adapted to other databases

- TS=fieldcode, including words from title, abstract, author keyword, and WoS applied keywords.
- NEAR/n = Proximity operator

(TS=("neet" OR "neets" OR "not in education, employment or training" OR "not in education, employment, or training" OR "Not in Employment, Education or Training" OR "Not participating in education, employment or training" OR "Not being integrated into education, employment, or training" OR "not engaged in education, employment or training" OR "Neither In Employment nor In Education or Training" OR "Neither Work nor Study" OR "neither study nor work" OR "Neither studying nor working" OR "neither in active employment nor in education and training" OR "neither in employment nor education and training" OR "neither in employment nor education and training" OR "neither work nor school" OR "neither in employment nor in education" OR "neither work nor enroll in postsecondary education" OR "neither employment nor training" OR "neither at school nor at employment" OR "neither work nor continue their education" OR "Neither education nor employment" OR "neither in school, working, nor seeking employment" OR "neither in school nor in the work force" OR "neither education, employment nor training" OR "neither employment nor education" OR "neither in school nor work*" OR "not studying, not working" OR "neither working nor attending school" OR "neither employment nor education*" OR "neither work nor education" OR "Neither Employment, Education nor Training" OR "neither working nor going to school" OR "neither worked nor studied" OR "neither work nor education" OR "neither education nor work*" OR "not in employment, education, or daily activities" OR "not being in education, employment, or training" OR "not in education, employment and training" OR "not in school, not employed" OR "not in education or employment" OR "not in education or work*" OR "not study and do not work" OR "neither studies nor works" OR "Neither in Employment, Education, nor Training" OR "Neither in Employment, Education, nor Training" OR "not in employment, education and training" OR "neither in education nor in employment or training" OR "neither in education nor in employment or training" OR "Not in Employment nor in Education or Training" OR "Not in Employment, nor in Education or Training" OR "neither in employment, education, or training" OR "neither working nor enrolled in school" OR "neither in employment, education, or training" OR "neither working nor in school" OR "neither been working nor studying") OR TS=((Young* OR youth* OR adolescen* OR teenage* OR juvenil*) NEAR/2 ("not" OR "nor" OR neither* OR "out of" OR outside* OR excluded) NEAR/2 (school* OR training OR educat* OR studying OR study OR employ* OR work* OR job* OR labour* OR labor*)) OR TI=((Young* OR youth* OR adolescen* OR teenage* OR juvenil*) AND (unemploy* OR un-employ* OR employ* OR re-employ* OR reemploy* OR labour* OR labor*))) AND TS=(randomi* OR randomly OR ((allocat* OR sample* OR assignment*) NEAR/2 random*) OR trial* OR intervention* OR program* OR effect* OR "control* group*" OR "control* stud*" OR "experimental* group*" OR "quasi experimental*" OR "outcome assessment*" OR subgroup* OR "Experimental design*" OR "Experimental study*" OR "control subject*")

Editions: WOS.SCI,WOS.SSCI,WOS.ESCI
Date Run: Tue Nov 28 2023 10:20:07 GMT+0100 (sentraleuropeisk normaltid)
Results: 3087

Link, Access is needed https://www.webofscience.com/wos/woscc/summary/3d21465d-e11a-41b7-b445-739c08e36ff4-b884d6fb/relevance/1

Scopus, Search date: 28-nov-2023. Result: 4795

(Book chapters, or conference abstracts are not excluded in the search strategy)

Copy search string, run from the Advanced search screen.

(TITLE-ABS-KEY-AUTH("neet" OR "neets" OR "not in education, employment or training" OR "not in education, employment, or training" OR "Not in Employment, Education or Training" OR "Not participating in education, employment or training" OR "Not being integrated into education, employment, or training" OR "not engaged in education, employment or training" OR "Neither In Employment nor In Education or Training" OR "Neither Work nor Study" OR "neither study nor work" OR "Neither studying nor working" OR "neither in active employment nor in education and training" OR "neither in employment nor education and training" OR "neither in employment nor education and training" OR "neither work nor school" OR "neither in employment nor in education" OR "neither work nor enroll in postsecondary education" OR "neither employment nor training" OR "neither at school nor at employment" OR "neither work nor continue their education" OR "Neither education nor employment" OR "neither in school, working, nor seeking employment" OR "neither in school nor in the work force" OR "neither education, employment nor training" OR "neither employment nor education" OR "neither in school nor work*" OR "not studying, not working" OR "neither working nor attending school" OR "neither employment nor education*" OR "neither work nor education" OR "Neither Employment, Education nor Training" OR "neither working nor going to school" OR "neither worked nor studied" OR "neither work nor education" OR "neither education nor work*" OR "not in employment, education, or daily activities" OR "not being in education, employment, or training" OR "not in education, employment and training" OR "not in school, not employed" OR "not in education or employment" OR "not in education or work*" OR "not study and do not work" OR "neither studies nor works" OR "Neither in Employment, Education, nor Training" OR "Neither in Employment, Education, nor Training" OR "not in employment, education and training" OR "neither in education nor in employment or training" OR "neither in education nor in employment or training" OR "Not in Employment nor in Education or Training" OR "Not in Employment, nor in Education or Training" OR "neither in employment, education, or training" OR "neither working nor enrolled in school" OR "neither in employment, education, or training" OR "neither working nor in school" OR "neither been working nor studying") OR TITLE-ABS-KEY-AUTH(((Young* OR youth* OR adolescen* OR teenage* OR juvenil*) w/2 ("not" OR "nor" OR neither* OR "out of" OR outside* OR excluded) w/2 (school* OR training OR educat* OR studying OR study OR employ* OR work* OR job* OR labour* OR labor*))) OR TITLE((Young* OR youth* OR adolescen* OR teenage* OR juvenil*) AND (unemploy* OR un-employ* OR employ* OR re-employ* OR reemploy* OR labour* OR labor*))) AND TITLE-ABS-KEY-AUTH(randomi* OR randomly OR ((allocat* OR sample* OR assignment*) w/2 random*) OR trial* OR intervention* OR program* OR effect* OR "control* group*" OR "control* stud*" OR "experimental* group*" OR "quasi experimental*" OR "outcome assessment*" OR subgroup* OR "Experimental design*" OR "Experimental study*" OR "control subject*")

Ovid databases: EBM Reviews - Cochrane Central Register of Controlled Trials <October 2023>; Embase <1974 to 2023 Week 47>; Ovid MEDLINE(R) ALL <1946 to November 27, 2023>; APA PsycInfo <1806 to November Week 2 2023> **Search date: Nov. 28, 2023**

Notes:

- ADJn = adjacency (proximity operator. Adj3 = NEAR/2 used in WoS)
- Field codes
    - ti,ab words from title or abstract, word indexed
    - kf (used in MEDLINE, EMBASE), author keyword
    - id (used in APA Psycinfo) The Key Concepts (ID) field concisely summarizes a document's subject content. Indexers use the Key Concepts to supplement Subject Headings (SH). Word indexed
    - pt publication type, phrase indexed. APA PsycInfo codes:
        - 0200 Book
        - 0240 Authored Book
        - 0280 Edited Book
        - 0300 Encyclopedia
        - 0400 Dissertation Abstract
    - hw = subject heading word, words from the database controlled vocabulary (Subject heading) word indexed. (Notice for the database Cochrane... the he HW index also includes words from the Keyword (KW) index and can serve as a single access point for searching MEDLINE, EMBASE, and all other CCTR records that have subject terms provided)
    - / = Search exact subject heading
- use oemez = search only EMBASE 1974- In line 8
- use psyh = search only APA PsycInfo Iin line 9
- Note: TRIAL protocols are not excluded from the search strategy

#	Query	Results from 28 Nov 2023
1	("neet" or "neets" or "not in education, employment or training" or "not in education, employment, or training" or "Not in Employment, Education or Training" or "Not participating in education, employment or training" or "Not being integrated into education, employment, or training" or "not engaged in education, employment or training" or "Neither In Employment nor In Education or Training" or "Neither Work nor Study" or "neither study nor work" or "Neither studying nor working" or "neither in active employment nor in education and training" or "neither in employment nor education and training" or "neither in employment nor education and training" or "neither work nor school" or "neither in employment nor in education" or "neither work nor enroll in postsecondary education" or "neither employment nor training" or "neither at school nor at employment" or "neither work nor continue their education" or "Neither education nor employment" or "neither in school, working, nor seeking employment" or "neither in school nor in the work force" or "neither education, employment nor training" or "neither employment nor education" or "neither in school nor work*" or "not studying, not working" or "neither working nor attending school" or "neither employment nor education*" or "neither work nor education" or "Neither Employment, Education nor Training" or "neither working nor going to school" or "neither worked nor studied" or "neither work nor education" or "neither education nor work*" or "not in employment, education, or daily activities" or "not being in education, employment, or training" or "not in education, employment and training" or "not in school, not employed" or "not in education or employment" or "not in education or work*" or "not study and do not work" or "neither studies nor works" or "Neither in Employment, Education, nor Training" or "Neither in Employment, Education, nor Training" or "not in employment, education and training" or "neither in education nor in employment or training" or "neither in education nor in employment or training" or "Not in	927

	Employment nor in Education or Training" or "Not in Employment, nor in Education or Training" or "neither in employment, education, or training" or "neither working nor enrolled in school" or "neither in employment, education, or training" or "neither working nor in school" or "neither been working nor studying").ti,ab,id,kf.	
2	neet*.hw.	75
3	((Young* or youth* or adolescen* or teenage* or juvenil*) adj3 ("not" or "nor" or neither* or "out of" or outside* or excluded) adj3 (school* or training or educat* or studying or study or employ* or work* or job* or labour* or labor*)).ti,ab,kf,id.	4,710
4	((Young* or youth* or adolescen* or teenage* or juvenil*) and (unemploy* or un-employ* or employ* or re-employ* or reemploy* or labour* or labor*)).ti.	5,585
5	or/1-4	10,821
6	(randomi* or randomly or ((allocat* or sample* or assignment*) adj3 random*) or trial* or intervention* or program* or effect* or "control* group*" or "control* stud*" or "experimental* group*" or "quasi experimental*" or "outcome assessment*").ti,ab,kf,id,hw. or treatment outcome/	36,871,035
7	5 and 6	6,263
8	Conference Abstract.pt. use oemez	4,960,334
9	("0400" or "02*" or "0300").pt. use psyh	1,097,688
10	or/8-9	6,058,022
**11**	**7 not 10**	**5,435**
12	***remove duplicates from 11***	***3,183***

Results from databases:

7 not 10	5435
EBM Reviews - Cochrane Central Register of Controlled Trials <October 2023>	257
Embase <1974 to 2023 Week 47>	2081
Ovid MEDLINE(R) ALL <1946 to November 27, 2023>	1750
APA PsycInfo <1806 to November Week 2 2023>	1347

Link, access is needed

https://ovidsp.ovid.com/ovidweb.cgi?T=JS&NEWS=N&PAGE=main&SHAREDSEARCHID=6m2myQk1XzaFJhzO7zt8I1gcfBsPIMMDHpclXJSuvXuNiIlA7Ed8POBZs2OGZnGLJ

CINAHL (EBSCO*host*).

Search modes - Boolean/Phrase. Interface - EBSCO*host* Research Databases, Search Screen - Advanced Search. Database – CINAHL

- MH = CINAHL Exact Subject Headings, [Phrase Indexed] Searches the exact CINAHL subject heading; searches both major and minor headings. May use qualifier abbreviations (also captures tertiary headings) or spelled with formatted dashes (truncate to capture items with tertiary headings) MH + explode the search term, thus includes any narrowing subject headings
- N# = Proximity
- CINAHL is not able to handle this phrase correct, "not studying, not working", thus leaved out in seacrh number S1:

#	Query	Limiters/Expanders	Results Nov. 28, 2023
S1	TI ("neet" OR "neets" OR "not in education, employment or training" OR "Not in Employment, Education or Training" OR "Not participating in education, employment or training" OR "Not being integrated into education, employment, or training" OR "not engaged in education, employment or training" OR "Neither In Employment nor In Education or Training" OR "Neither Work nor Study" OR "neither study nor work" OR "Neither studying nor working" OR "neither in active employment nor in education and training" OR "neither in employment nor education and training" OR "neither in employment nor education and training" OR "neither work nor school" OR "neither in employment nor in education" OR "neither work nor enroll in postsecondary education" OR "neither employment nor training" OR "neither at school nor at employment" OR "neither work nor continue their education" OR "Neither education nor employment" OR "neither in school, working, nor seeking employment" OR "neither in school nor in the work force" OR "neither education, employment nor training" OR "neither employment nor education" OR "neither in school nor work*" OR "neither working nor attending school" OR "neither employment nor education*" OR "neither work nor education" OR "Neither Employment, Education nor Training" OR "neither working nor going to school" OR "neither worked nor studied" OR "neither work nor education" OR "neither education nor work*" OR "not in employment, education, or daily activities" OR "not being in education, employment, or training" OR "not in education, employment and training" OR "not in school, not employed" OR "not in education or employment" OR "not in education or work*" OR "not study and do not work" OR "neither studies nor works" OR "Neither in Employment, Education, nor Training" OR "Neither in Employment, Education, nor Training" OR "not in employment, education and training" OR "neither in education nor in employment or training" OR "neither in education nor in employment or training" OR "Not in Employment nor in Education or Training" OR "Not in Employment, nor in Education or Training" OR "neither in employment, education, or training" OR "neither working nor enrolled in school" OR "neither in employment, education, or training" OR "neither working nor in school" OR "neither been working nor studying")		62
S2	AB ("neet" OR "neets" OR "not in education, employment or training" OR "Not in Employment, Education or Training" OR		215

	"Not participating in education, employment or training" OR "Not being integrated into education, employment, or training" OR "not engaged in education, employment or training" OR "Neither In Employment nor In Education or Training" OR "Neither Work nor Study" OR "neither study nor work" OR "Neither studying nor working" OR "neither in active employment nor in education and training" OR "neither in employment nor education and training" OR "neither in employment nor education and training" OR "neither work nor school" OR "neither in employment nor in education" OR "neither work nor enroll in postsecondary education" OR "neither employment nor training" OR "neither at school nor at employment" OR "neither work nor continue their education" OR "Neither education nor employment" OR "neither in school, working, nor seeking employment" OR "neither in school nor in the work force" OR "neither education, employment nor training" OR "neither employment nor education" OR "neither in school nor work*" OR "neither working nor attending school" OR "neither employment nor education*" OR "neither work nor education" OR "Neither Employment, Education nor Training" OR "neither working nor going to school" OR "neither worked nor studied" OR "neither work nor education" OR "neither education nor work*" OR "not in employment, education, or daily activities" OR "not being in education, employment, or training" OR "not in education, employment and training" OR "not in school, not employed" OR "not in education or employment" OR "not in education or work*" OR "not study and do not work" OR "neither studies nor works" OR "Neither in Employment, Education, nor Training" OR "Neither in Employment, Education, nor Training" OR "not in employment, education and training" OR "neither in education nor in employment or training" OR "neither in education nor in employment or training" OR "Not in Employment nor in Education or Training" OR "Not in Employment, nor in Education or Training" OR "neither in employment, education, or training" OR "neither working nor enrolled in school" OR "neither in employment, education, or training" OR "neither working nor in school" OR "neither been working nor studying")		
S3	TI (((Young* OR youth* OR adolescen* OR teenage* OR juvenil*) N2 ("not" OR "nor" OR neither* OR "out of" OR outside* OR excluded) N2 (school* OR training OR educat* OR studying OR study OR employ* OR work* OR job* OR labour* OR labor*))) OR TI ((Young* OR youth* OR adolescen* OR teenage* OR juvenil*) AND (unemploy* OR un-employ* OR employ* OR re-employ* OR reemploy* OR labour* OR labor*))		1,052
S4	AB (((Young* OR youth* OR adolescen* OR teenage* OR juvenil*) N2 ("not" OR "nor" OR neither* OR "out of" OR outside* OR excluded) N2 (school* OR training OR educat* OR studying OR study OR employ* OR work* OR job* OR labour* OR labor*)))		421
S5	S1 OR S2 OR S3 OR S4		1,610
S6	TI ((randomi* OR randomly OR ((allocat* OR sample* OR assignment*) N2 random*) OR trial* OR intervention* OR program* OR effect* OR "control* group*" OR "control* stud*" OR "experimental* group*" OR "quasi experimental*" OR "outcome assessment*" OR subgroup* OR "Experimental design*" OR "Experimental study*" OR "control subject*"))		811,915
S7	AB ((randomi* OR randomly OR ((allocat* OR sample* OR assignment*) N2 random*) OR trial* OR intervention* OR program* OR effect* OR "control* group*" OR "control* stud*"		2,004,475

	OR "experimental* group*" OR "quasi experimental*" OR "outcome assessment*" OR subgroup* OR "Experimental design*" OR "Experimental study*" OR "control subject*"))		
S8	(MH "Experimental Studies+") OR (MH "Treatment Outcomes")		745,472
S9	S6 OR S7 OR S8		2,603,690
S10	S5 AND S9		838
S11	***S5 AND S9***	***Limiters - Peer Reviewed***	***812***

ERIC (EBSCO*host*). Search date Nov. 28, 2023

Search modes - Boolean/Phrase

Notes

- Field code: DE - Subjects, [Phrase Indexed], Searches exact subject heading descriptors.
- Search 1: The phrases for NEET are not included in the search strategy for ERIC. The database is not able to handle the search string correctly, so only the abbreviations for NEET OR NEETs are included.

**#**	**Query**	**Limiters/Expanders**	**Results, Nov. 28, 2023**
S1	TI ("neet" OR "neets") OR TI ((Young* OR youth* OR adolescen* OR teenage* OR juvenil*) AND (unemploy* OR un-employ* OR employ* OR re-employ* OR reemploy* OR labour* OR labor*))		1,719
S2	AB ("neet" OR "neets")		142
S3	TI (((Young* OR youth* OR adolescen* OR teenage* OR juvenil*) N2 ("not" OR "nor" OR neither* OR "out of" OR outside* OR excluded) N2 (school* OR training OR educat* OR studying OR study OR employ* OR work* OR job* OR labour* OR labor*)))		177
S4	AB (((Young* OR youth* OR adolescen* OR teenage* OR juvenil*) N2 ("not" OR "nor" OR neither* OR "out of" OR outside* OR excluded) N2 (school* OR training OR educat* OR studying OR study OR employ* OR work* OR job* OR labour* OR labor*)))		874
S5	S1 OR S2 OR S3 OR S4		2,688
S6	TI ((randomi* OR randomly OR ((allocat* OR sample* OR assignment*) N2 random*) OR trial* OR intervention* OR program* OR effect* OR "control* group*" OR "control* stud*" OR "experimental* group*" OR "quasi experimental*" OR "outcome assessment*" OR subgroup* OR "Experimental design*" OR "Experimental study*" OR "control subject*"))		211,022
S7	AB ((randomi* OR randomly OR ((allocat* OR sample* OR assignment*) N2 random*) OR trial* OR intervention* OR program* OR effect* OR "control* group*" OR "control* stud*" OR "experimental* group*" OR "quasi experimental*" OR "outcome assessment*" OR subgroup* OR "Experimental design*" OR "Experimental study*" OR "control subject*"))		768,992
S8	DE "Randomized Controlled Trials" OR DE "Control Groups" OR DE "Experimental Groups" OR DE "Matched Groups" OR DE "Effect Size"		21,044
S9	S6 OR S7 OR S8		807,612
S10	**S5 AND S9**		**1,658**
S11	***S5 AND S9***	***Limiters - Peer Reviewed***	***494***

SocINDEX (EBSCO*host*). Search date, Nov. 28, 2023

Search modes - Boolean/Phrase. Notes as ERIC above

#	Query	Limiters/Expanders	Results, Nov. 28, 2023
S1	TI (((Young* OR youth* OR adolescen* OR teenage* OR juvenil*) N2 ("not" OR "nor" OR neither* OR "out of" OR outside* OR excluded) N2 (school* OR training OR educat* OR studying OR study OR employ* OR work* OR job* OR labour* OR labor*)))		68
S2	AB (((Young* OR youth* OR adolescen* OR teenage* OR juvenil*) N2 ("not" OR "nor" OR neither* OR "out of" OR outside* OR excluded) N2 (school* OR training OR educat* OR studying OR study OR employ* OR work* OR job* OR labour* OR labor*)))		432
S3	DE "YOUNG people not in education, employment, or training"		33
S4	TI ((Young* OR youth* OR adolescen* OR teenage* OR juvenil*) AND (unemploy* OR un-employ* OR employ* OR re-employ* OR reemploy* OR labour* OR labor*))		2,040
S5	S1 OR S2 OR S3 OR S4		2,504
S6	TI ((randomi* OR randomly OR ((allocat* OR sample* OR assignment*) N2 random*) OR trial* OR intervention* OR program* OR effect* OR "control* group*" OR "control* stud*" OR "experimental* group*" OR "quasi experimental*" OR "outcome assessment*" OR subgroup* OR "Experimental design*" OR "Experimental study*" OR "control subject*"))		150,784
S7	AB ((randomi* OR randomly OR ((allocat* OR sample* OR assignment*) N2 random*) OR trial* OR intervention* OR program* OR effect* OR "control* group*" OR "control* stud*" OR "experimental* group*" OR "quasi experimental*" OR "outcome assessment*" OR subgroup* OR "Experimental design*" OR "Experimental study*" OR "control subject*"))		647,301
S8	DE "CLINICAL trials" OR DE "RANDOMIZED controlled trials"		8,863
S9	S6 OR S7 OR S8		683,284
**S10**	**S5 AND S9**		**917**
S11	***S5 AND S9***	***Limiters - Peer Reviewed***	***819***
