# Supplementary material for: Interventions targeting young people not in employment, education or training (NEET) for increased likelihood of return to school or employment—A systematic review
Source: PLoS One. 2024 Jun 27;19(6):e0306285. doi: 10.1371/journal.pone.0306285 (PMC11210863; doi:10.1371/journal.pone.0306285)
Supplement: S3 File — (PDF) [file pone.0306285.s003.pdf]

Number	Author	Title	Main reason for exclusion
1.	Bal et al., 2023 (1)	Effect evaluation of a vocational rehabilitation program for young adults with chronic physical conditions at risk for unemployment: a controlled clinical trial	Wrong population
2.	Creed et al., 1996 (2)	Neuroticism and mental health outcomes for long-term unemployed youth attending occupational skills training programs	Wrong population
3.	Ekstrom et al., 1987 (3)	The effects of youth employment program participation on later employment	Wrong population
4.	Ferguson, 2018 (4)	Employment outcomes from a randomized controlled trial of two employment interventions with homeless youth	Wrong population
5.	Ferguson, 2018 (5)	Nonvocational outcomes from a randomized controlled trial of two employment interventions for homeless youth	Wrong population
6.	Ibarraran et al., 2014 (6)	Life skills, employability and training for disadvantaged youth: evidence from a randomized evaluation design	Wrong population
7.	Leathers et al., 2023 (7)	Mentoring, employment assistance, and enhanced staff outreach for older youth in care: outcomes from a randomized controlled trial	Wrong population
8.	Lindsay et al., 2019 (8)	A 4-week electronic-mentoring employment intervention for youth with physical disabilities: Pilot randomized controlled trial	Wrong population
9.	Maree et al., 2019 (9)	Using career counselling with group life design principles to improve the employability of disadvantaged young adults	Wrong population
10.	Park et al., 2020 (10)	An evaluation of the youth employment support program in South Korea: focusing on the outcome of preventing NEET	Wrong population
11.	Santini et al., 2020 (11)	Older adult entrepreneurs as mentors of young people neither in employment nor education and training (NEETs). Evidences from multi-country intergenerational learning program	Wrong population

12.	Tandon et al., 2015 (12)	Depression outcomes associated with an intervention implemented in employment training programs for low-income adolescents and young adults	Wrong population
13.	Jackson et al., 1986 (13)	A success story for the summer youth employment training program	Wrong study design
14.	Ngai et al., 2023 (14)	Career and life development intervention for non-engaged youth: evaluating the Hong Kong Benchmarks (Community) pilot program	Wrong population
15.	Jennings Mayo-Wilson et al., 2020 (15)	Acceptability of a feasibility randomized clinical trial of a microenterprise intervention to reduce sexual risk behaviors and increase employment and HIV preventive practices (EMERGE) in young adults: a mixed methods assessment	Wrong study design
16.	Morton et al., 2012 (16)	Empowerment-based non-formal education for Arab youth: a pilot randomized trial	Wrong population
17.	Park et al., 2021 (17)	Effects of an alcohol-related harm prevention program among out-of-school female adolescents	Wrong outcome
18.	Das et al., 2021 (18)	Training the disadvantaged youth and labor market outcomes: evidence from Bangladesh	Wrong population
19.	Donovan et al., 1986 (19)	Employment status and psychological well-being – a longitudinal study of 16-year-old school leavers	Wrong population
20.	Attanasio et al., 2011 (20)	Subsidizing vocational training for disadvantaged youth in Colombia: evidence from a randomized trial	Wrong population
21.	Borland et al., 2013 (21)	Does coordination of welfare services delivery make a difference for extremely disadvantaged jobseekers? Evidence from the 'YP4' trial	Wrong study design
22.	Borland et al., 2007 (22)	Does a minimum job search requirement reduce time on unemployment payments? Evidence from the jobseeker diary in Australia	Wrong study design

References

1. Bal MI, Hilberink SR, Roelofs P, van der Slot WMA, Bentvelsen L, Miedema HS, et al. Effect evaluation of a vocational rehabilitation program for young adults with chronic physical conditions at risk for unemployment: A controlled clinical trial. Scand J Occup Ther. 2023;30(8):1292-302.
2. Creed PA, Machin MA, Hicks R. Neuroticism and mental health outcomes for long-term unemployed youth attending occupational skills training programs. Personality and Individual Differences. 1996;21(4):537-44.
3. The effects of youth employment program participation on later employment, (1987).
4. Ferguson KM. Employment Outcomes From a Randomized Controlled Trial of Two Employment Interventions With Homeless Youth. J Soc Social Work Res. 2018;9(1):1-21.
5. Ferguson KM. Nonvocational Outcomes From a Randomized Controlled Trial of Two Employment Interventions for Homeless Youth. Research on Social Work Practice. 2018;28(5):603-18.
6. Ibarraran P, Ripani L, Taboada B, Villa JM, Garcia B. Life skills, employability and training for disadvantaged youth: Evidence from a randomized evaluation design. IZA Journal of Labor & Development. 2014;3(1):10.
7. Leathers SJ, Holtschneider C, Ludington M, Ross EV, Barnett JL. Mentoring, employment assistance, and enhanced staff outreach for older youth in care: Outcomes from a randomized controlled trial. Children and Youth Services Review. 2023;153:107095.
8. Lindsay S, Cagliostro E, Stinson J, Leck J. A 4-Week Electronic-Mentoring Employment Intervention for Youth With Physical Disabilities: Pilot Randomized Controlled Trial. JMIR Pediatr Parent. 2019;2(1):e12653.
9. Maree JG, Gerryts EW, Fletcher L, Olivier J. Using career counselling with group life design principles to improve the employability of disadvantaged young adults. Journal of Psychology in Africa. 2019;29(2):110-20.
10. Park M, Lee S, Nam KC, Noh H, Lee S, Lee BJ. An evaluation of the youth employment support program in South Korea: Focusing on the outcome of preventing NEET. Children and Youth Services Review. 2020;110:104747.
11. Santini S, Baschiera B, Socci M. Older adult entrepreneurs as mentors of young people neither in employment nor education and training (NEETs). Evidences from multi-country intergenerational learning program. Educational Gerontology. 2020;46(3):97-116.
12. Tandon SD, Latimore AD, Clay E, Mitchell L, Tucker M, Sonenstein FL. Depression outcomes associated with an intervention implemented in employment training programs for low-income adolescents and young adults. JAMA Psychiatry. 2015;72(1):31-9.
13. Jackson LS, Whitten AY. A SUCCESS STORY FOR THE SUMMER YOUTH EMPLOYMENT TRAINING PROGRAM. Community Junior College Research Quarterly of Research and Practice. 1986;10(1):13-22.
14. Ngai SS-y, Cheung C-k, Zhou Q, Wang L, Ng Y-h, Leung WP-c, et al. Career and life development intervention for non-engaged youth: Evaluating the Hong Kong Benchmarks (Community) Pilot Program. Frontiers in Psychology. 2023;14.
15. Jennings Mayo-Wilson L, Coleman J, Timbo F, Latkin C, Torres Brown ER, Butler AI, et al. Acceptability of a feasibility randomized clinical trial of a microenterprise intervention to reduce sexual risk behaviors and increase employment and HIV preventive practices (EMERGE) in young adults: a mixed methods assessment. BMC Public Health. 2020;20(1):1846.
16. Morton MH, Montgomery P. Empowerment-based non-formal education for Arab youth: A pilot randomized trial. Elsevier Science; 2012. p. 417-25.

17. Park H, Kim S, Yang J. Effects of an Alcohol-Related Harm Prevention Program among Out-of-School Female Adolescents. Int J Environ Res Public Health. 2021;18(8).
18. Das N. Training the disadvantaged youth and labor market outcomes: Evidence from Bangladesh. Journal of Development Economics. 2021;149:102585.
19. Donovan A, Oddy M, Pardeo R, Ades A. EMPLOYMENT STATUS AND PSYCHOLOGICAL WELL-BEING: A LONGITUDINAL STUDY OF 16-YEAR-OLD SCHOOL LEAVERS. Journal of Child Psychology and Psychiatry. 1986;27(1):65-76.
20. Attanasio O, Kugler A, Meghir C. Subsidizing Vocational Training for Disadvantaged Youth in Colombia: Evidence from a Randomized Trial. American Economic Journal: Applied Economics. 2011;3(3):188-220.
21. Borland J, Tseng Y-P, Wilkins R. Does Coordination of Welfare Services Delivery Make a Difference for Extremely Disadvantaged Jobseekers? Evidence from the ‘YP4’ Trial. Economic Record. 2013;89(287):469-89.
22. Borland J, Tseng Y-P. Does a Minimum Job Search Requirement Reduce Time on Unemployment Payments? Evidence from the Jobseeker Diary in Australia. ILR Review. 2007;60(3):357-78.
